# Study of the Oxidative and Microbiological Stability of Nitrite-Reduced, Vacuum-Packed, Refrigerated Lamb Sausages Supplemented with Red Propolis Extract

**DOI:** 10.3390/foods12244419

**Published:** 2023-12-09

**Authors:** Luciana Ruggeri Menezes Gotardo, Francisco Allan Leandro de Carvalho, Dannaya Julliethy Gomes Quirino, Carmen Sílvia Favaro-Trindade, Severino Mathias de Alencar, Alessandra Lopes de Oliveira, Marco Antonio Trindade

**Affiliations:** 1 School of Animal Science and Food Engineering, University of Sao Paulo (USP), Pirassununga 13635-900, SP, Brazil; luciana.ruggeri@usp.br (L.R.M.G.); carmenft@usp.br (C.S.F.-T.);; 2 Department of Animal Nutrition, Agricultural Sciences Campus, Federal University of Vale do São Francisco, Petrolina 56304-917, PE, Brazil; francisco.allan@univasf.edu.br; 3 Department of Agri-Food Industry, Food, and Nutrition, Luiz de Queiroz College of Agriculture, University of Sao Paulo (USP), Piracicaba 13418-900, SP, Brazil

**Keywords:** lamb meat, natural antioxidants, lipid oxidation, psychrotrophic microorganisms, sensory acceptance

## Abstract

Vacuum-packed lamb sausages with or without red propolis extract and a reduced sodium nitrite content were evaluated for oxidative and microbiological stability during storage for 21 days at 2 °C. The following treatments were evaluated: EN150 (control, base formulation (BF) + 500 mg/kg sodium erythorbate and 150 mg/kg sodium nitrite); EN75 (BF + 500 mg/kg sodium erythorbate and 75 mg/kg sodium nitrite); P1N75 (without the addition of erythorbate, BF + 1800 mg/kg propolis extract and 75 mg/kg sodium nitrite); and P2N75 (without the addition of erythorbate, BF + 3600 mg/kg propolis extract and 75 mg/kg sodium nitrite). Analyses were conducted to characterize the samples on day 0 with respect to the proximate composition (moisture, protein, fat, and ash) and sensory acceptance. Stability during refrigerated storage was evaluated on days 0, 7, 14 and 21 for the parameters pH, color profile (L*, a*, and b*), TBARs index (oxidative stability) and microbiological count of aerobic psychrotrophic microorganisms. Texture profile, cooking weight loss (WLC), peroxide index and free fatty acids were evaluated on days 0 and 21. The treatments with propolis and reduced nitrite (EN150 and P1N75) showed a red color intensity (a*) similar to the treatment with erythorbate and the same nitrite content (EN75) at the end of storage, maintaining the characteristic reddish color of the sausages. The extract slowed down lipid oxidation during storage, especially P2N75, which showed the lowest level of TBARS (0.39 mg MDA/kg) and the peroxide index (2.13 mEq g O_2_) on day 21. The residual nitrite value in EN75 was the lowest (*p* < 0.05) on day 21, showing that synthetic antioxidants are more efficient than the extract in nitrite reduction reactions. The results for the counts of psychrotrophic microorganisms showed that the extract did not have the expected antimicrobial effect on the growth of this microorganisms, and leveling the results revealed no differences (*p* < 0.05) between the treatments. Despite the red propolis extract not showing a significant antimicrobial improvement in lamb sausages, it can be considered a healthy option with good prospects for replacing synthetic antioxidants with a natural product.

## 1. Introduction

Processed meats are highly susceptible to losing their characteristic color, losing the functionality of lipids/proteins, and developing a rancid taste due to the reagents formed during oxidation. Oxidative reactions in meat products are the main focus of researchers in meat preservation, due to their role in deteriorating quality and generating toxic compounds combined with an altered aroma and flavor, as well as causing discoloration of the meat, which consequently leads to rejection by consumers, making it necessary to add antioxidants to these foods [1,2]. However, the evolution in consumer awareness of the possible harmful effects on health caused by synthetic additives has led to an increase in the search for foods with a healthier appeal. In this sense, several studies have confirmed the fundamental role of ingredients of plant origin and their bioactive components, such as polyphenols and flavonoids, in preventing lipid oxidation by inhibiting the formation of free radicals in food [3].

Propolis is a product with anti-inflammatory, antimicrobial [4], anticarcinogenic [5] and antioxidant [6] potential, and its properties make it an interesting material for use in the food industry. The most prevalent isolated compounds in propolis are flavonoids, which are directly related to its intense red color and potential biological action [7]. Although several studies have focused on investigating the biological effects of the activity of red propolis, the role of this resin in restructured meat products and sausages is little known still, and it could therefore be an excellent alternative for use in the preparation of functional foods [8]. Nitrite is added to fresh meat sausages with the aim of inhibiting the growth of some microorganisms, reducing the possibility of oxidative rancidity and maintaining the pink color and characteristic flavor [9]. Its presence in the human body is highly reactive in an acidic environment, such as the stomach [10], and it forms endogenous N-nitroso compounds (NOCs). These compounds can circulate in the human body [11], causing diseases such as methemoglobinemia, which is a potentially fatal condition in which less oxygen than usual is supplied to the body’s cells [10]. In addition, another negative point associated with nitrite is the formation of N-nitrosamines during preparation at home and in the gastrointestinal tract after consumption of these meat derivatives, [12] most of which are classified as possibly carcinogenic [13]. Therefore, a reduction in the use of sodium nitrite has been practiced due to the potential risks it poses to human health. Thus, the addition of reduced levels of nitrite associated with natural antioxidants is a trend in research and consumer markets. This is due to the presence, in natural products such as red propolis, of phenolic compounds that have the ability to inhibit or reduce lipid and microbial oxidation in meat products [14].

Therefore, this study aimed to evaluate how the bioactive compounds present in red propolis extract act as antimicrobials and antioxidants together with nitrite and sodium erythorbate by verifying their effects on the physicochemical, microbiological and sensory characteristics of vacuum-packed lamb sausages stored under refrigeration at 2 °C.

## 2. Materials and Methods

### 2.1. Red Propolis Extract

The raw material used to produce the red propolis extract was purchased in the northeast of Brazil. The red propolis was crushed in a blender (model RI 1710, Walita, São Paulo, Brazil) to obtain smaller fragments and then used to prepare the extract. The extraction methodology used was that proposed by [15], with modifications. The ethanolic extract was prepared using a ratio of 30 g of propolis to 100 mL of 80% ethanol in a water bath at 50 °C with mechanical stirring at 800 rpm for 30 min. The samples were then centrifuged at 25 °C and 4500× *g* for 10 min in order to remove solid dirt and obtain the ethanolic extract. This process was finalized after passing the samples through a rotaevaporator (TE-211, Tecnal, Piracicaba, Brazil) at a temperature of 45 °C, concentrating them to 40% of the initial volume. The extraction steps were conducted while avoiding exposure to light.

### 2.2. Determination of the Antioxidant Capacity of the Red Propolis Extract

#### 2.2.1. Determination of the Total Phenolic Content

The total phenolic content was determined as described by [16], with some modifications. The standard used was gallic acid, and the total phenolic content was determined using Folin-Ciocalteu reagent. Appropriate dilutions of red propolis extract were prepared with water (0.5 mL) and added to 2.5 mL of Folin-Ciocalteu reagent (diluted 1:10 with water) and 2 mL of a Na_2_CO_3_ solution (7.5%). The reaction took place at 50 °C for 15 min, and the absorbance was measured at 760 nm. The total phenolic content was expressed as mg of gallic acid equivalent (GAE)/100 g of propolis extract.

#### 2.2.2. ABTS Radical Cation (ABTS) Decolorization Test

To determine the reducing capacity of the ABTS radical (2,2′-azino-bis (3-ethylbenzothiazolin) 6-acidosulfonic acid), the methodology according to [17] was used, after previously obtaining a calibration curve of 0.5 to 10.0 mL of a Trolox solution (6-hydroxy-2,5,7,8-tetramethylchromo-2-carboxylic acid). The ABTS* solution was prepared with a solution of potassium persulphate (140 mM) and ABTS (7 mM), and the reaction was allowed to proceed for 16 h under protection from light. The sample of propolis extract was prepared with 3.0 mL of the ABTS radical and 30.0 uL of the ethanolic extract of red propolis, followed by stirring and resting for 6 min in the absence of light. The absorbance was measured at 734 nm and the results were expressed as mmol of Trolox equivalent (mmol TE) per 100 mg of propolis extract.

#### 2.2.3. DPPH

The DPPH (1,1-diphenyl-2-picrylhydrazyl) elimination method was conducted with some modifications according to the procedure previously described by [18]. One hundred microliters of samples of propolis extract were added to 3900 μL of DPPH solution (60 μM in methanol). After an incubation at 37 °C for 10 min, absorbance readings were taken at 515 nm. The DPPH scavenging activity of the extracts was determined using Trolox as a standard, and the results were expressed as mg of Trolox/g of propolis extract.

### 2.3. Sausage Formulation and Processing

The fresh sausages were produced in the Meat Quality and Stability Laboratory, Faculty of Food Engineering and Animal Science, Pirassununga Campus, USP (University of São Paulo).

The raw material used was the same and from the same batch in all treatments. All the formulations contained the following ingredients: lamb meat ground into an 8 mm disk (75%), pork bacon ground into a 6 mm disk (20%), salt (2.0%), onion (0.3%), garlic (0.1%) and pepper (0.1%) as the “Base Formulation” (BF), plus the ingredients/additives described below for each treatment.

Four treatments were processed, namely:

– EN150 (control, BF + 500 mg/kg sodium erythorbate and 150 mg/kg sodium nitrite);
– EN75 (BF + 500 mg/kg sodium erythorbate and 75 mg/kg sodium nitrite);
– P1N75 (without the addition of erythorbate, BF + 1800 mg/kg propolis extract and 75 mg/kg sodium nitrite);
– P2N75 (without the addition of erythorbate, BF + 3600 mg/kg propolis extract and 75 mg/kg sodium nitrite).

The whole experiment was repeated three times at the process level on three different dates. A total of 252 sample units of sausage weighing +/− 80 g each were produced, with 84 sausages per repetition (three process repetitions) and 21 sausages per treatment.

The use of sodium erythorbate as an antioxidant is a common practice in the meat product industry in Brazil, and the usual level for its addition is 500 ppm, which was the value established as the basis for establishing the equivalence of the propolis extract. It is worth noting that the amounts of propolis added were based on the antioxidant equivalence of the total phenolic value of erythorbate, with 500 mg being equivalent to 1800 mg of propolis. Therefore, the equivalence was used in one treatment (P1N75) and double that in the other treatment (P2N75). Regarding the EN150 and EN75 treatments, the same level of erythorbate (500 ppm) was maintained in both treatments in order to evidence only possible differences caused by nitrite reduction.

The ingredients were weighed, mixed and homogenized by hand, and the final mass was placed in a manual sausage maker. Natural pork casings were used to stuff the fresh sausages. The sausage links were vacuum-packed individually in plastic bags (poly-nylon, 0.16 mm thick), identified and stored at 2 °C in a BOD for later analysis. The following parameters were evaluated: pH, color profile, TBARS (oxidative stability), and microbiology at 0, 7, 14, and 21 days of storage. The texture profile, cooking weight loss, residual nitrite, peroxide, and free fatty acids were analyzed on days 0 and 21, while the proximate composition and sensory analyses were carried out only once.

### 2.4. Physico-Chemical Evaluation of Fresh Sausages

#### 2.4.1. Proximate Composition

The official [19] methodologies for determining moisture (950.46), fixed mineral residue (920.153), and protein (928.08) were used to determine and calculate the proximate composition. The lipid content was determined using the method in [20].

#### 2.4.2. pH and Color Profile

An insertion pH meter model Hanna^®^ HI 99163 peagameter (Hanna^®^ Instruments Inc. Woonsocket, RI, USA, made in Romania, RO) was used to read the pH of the sausages in triplicate. Beforehand, however, the pH meter was calibrated with pH 4 and 7 solutions. To obtain the color values, a Hunter-Lab colorimeter (Minolta, Osaka, Japan) was used, obtaining L*, a*, and b* values determined by the angle of observation of the D 65 illuminant in a 10 cell and an aperture of 30 mm. Readings were taken in quadruplicate.

#### 2.4.3. Weight Loss on Cooking

The percentage of weight loss due to cooking of the sausages was calculated according to [21]. The samples were stored at 2 °C, vacuum-packed and weighed raw. They were then baked in an oven at 180 °C with a temperature probe inserted into each one until the internal temperature reached 72 °C, then removed and cooled to an internal temperature of 25 °C. Triplicates were used for each treatment. The percentage loss was calculated according to the equation below:

% Weight loss to cooking = [(Initial mass − Final mass) × 100]/Initial mass

#### 2.4.4. Texture Profile (TPA)

These analyses were conducted using a TA.XT 2i Texturometer (Texture Analyzer, Stable Micro Systems, Godalming, UK) at 25 °C by performing compression tests with a P35 cylindrical probe at a constant speed of 2 mm/s and a time of 2 s between compressions up to a deformation of 50%. The hardness (N), elasticity (mm), cohesiveness, gumminess (kg × mm), chewiness (kg), and strength values were determined. After cooking in the oven, each sausage was individually cut into 5 cylindrical pieces, 2 cm high, totaling 15 samples from each treatment, and submitted for a determination of the texture profile according to [22].

#### 2.4.5. Lipid Oxidation (TBARS)

The oxidative stability of the samples was monitored using the method developed by [23], with modifications. This methodology consists of the reaction between oxidation products and thiobarbituric acid, resulting in compounds that were measured with a spectrophotometer at 534 nm. A solution of 1,1,3,3-tetraethoxypropane (TEP) was prepared and used as a standard for the standard curve, and the results were expressed as mg malonaldehyde equivalent (MDA)/kg of sample of sausage.

#### 2.4.6. Free Fatty Acids

The free fatty acid profile of the lamb sausages was determined on days 0 and 21 of storage from the lipid fraction extracted using the methodology described in [20]. The samples were weighed to 0.5 g in a 50 mL Falcon tube with a lid, which remained frozen in an ultra-freezer (−80 °C). The samples were then subjected to saponification, neutralization, separation of the fatty acid salts, and esterification. Finally, free fatty acids were determined and analyzed using gas chromatography-mass spectrometry (QP 2010 Plus, Shimadzu, Tokyo, Japan) with a split/splitless injector (AOC-500, Shimadzu, Tokyo, Japan) and an automatic injector (model AOC-500). The stationary phase was a capillary column (100 m × 0.25 mm di × 0.20 µm df, SP-2560 Supelco, Bellefonte, PA, USA) using helium gas as a carrier at a flow rate of 1.59 mL/min. The samples were injected into an injector with a split ratio of 1:40, a temperature of 250 °C, and a column pressure of 300 kpa according to [24].

#### 2.4.7. Determination of the Peroxide Index

The methodology used to determine the peroxide index was that of [25]. The fat samples extracted from sausages were weighed (1 ± 0.05 g), and 30 mL of the 1:1 acetic acid-chloroform solution was added under agitation, followed by 0.5 mL of the saturated KI solution, and the mixture was left to stand for five minutes. The solution was titrated, but first 30 mL of water and 0.5 mL of starch indicator solution were added with 0.1 N or 0.01 N sodium thiosulfate solution with constant stirring until the yellow color had almost disappeared. The results were expressed in mEq g/kg fat.

#### 2.4.8. Residual Nitrite

The methodology used to determine residual nitrite was that of the [26], number 283/IV - Spectrophotometric determination of nitrites. This is based on the diazotation reactions of nitrite with sulphanilic acid and copulation with alpha-naphthylamine hydrochloride in an acidic medium, forming pinkish alpha-naphthylamino-azobenzene-p-sulfonic acid. The resulting product was determined spectrophotometrically at 540 nm. The results were expressed in mg/kg of sample.

### 2.5. Microbiology Analysis

The microbiological analysis conducted on the sausages was for psychrotrophic aerobic microorganisms. Samples of 25 g were weighed aseptically and homogenized in 225 mL of 0.1% sterile peptone water in a laminar flow hood at room temperature. Serial decimal dilutions were prepared for each sample. The methodology used for aerobic psychrotrophs was APHA Standard 13.61.205 [27] in Petri dishes with standard counting agar (PCA) made by sowing on the surface, incubating the plates inverted at 17 °C for 16 h, followed by a further 72 h at 7 °C and then counting. Analyses were conducted on days 0, 7, 14, and 21.

Compact dry SL^®^ rapid test plates were used to analyze *Salmonella* sp. and Compact dry EC^®^ plates were used for *Escherichia coli*, for both of which 1 mL of the sample was inoculated and incubated inverted at 37 °C in an incubator for 24 h. These last analyses were conducted to guarantee and ensure the safety of the samples offered to the tasters who took part in the sensory analyses, so only the presence and absence of these microorganisms were read.

### 2.6. Sensory Analysis

The project was approved by the FZEA/USP Research Ethics Committee (CAAE 44670421.6.0000.5422). The method used was acceptance based on a nine-point hedonic scale (1 = I disliked it very much; 9 = I liked it very much), according to the methodology described in [28], in which each of the treatments was evaluated monadically. The consumers evaluated how much they liked the samples in terms of aroma, texture, juiciness, flavor, color, and overall quality. The acceptance analysis was conducted by 120 untrained consumers, 30% men and 70% women, aged between 18 and 58 years, with convenience sampling, in the city of Pirassununga on the USP-FZEA Campus. The only requirement for recruitment was that the consumers should like sheep meat products. The samples were prepared in the Sensory Analysis Laboratory, roasted in an electric oven until they reached 72 °C in the center of the piece, cut into pieces of +/− 20 g, wrapped in aluminum foil, and stored in an oven at 60 °C. The samples were served in disposable cups coded with three random digits. All care was taken with storage, transportation, and offering to the evaluator, guaranteeing the food safety of those involved.

### 2.7. Statistical Analysis

The statistical analyses conducted in this study were performed using the Statistical Analysis System (SAS version 9.4, SAS Institute INC., Cary, NC, USA). A normal distribution and homogeneity of variance were previously tested (Shapiro–Wilk). The data were submitted to analysis of variance (ANOVA), and when ANOVA showed a significant effect (*p* < 0.05), the results were evaluated using the Tukey test. For physicochemical analysis data, the treatments were considered as fixed effects and the manufacturing repetitions (every experiment was repeated three times) and storage times (0, 7, 14, and 21 days) as random effects. The statistics for the sensory analysis were conducted using the Kruskal–Wallis test for independent samples with the hypothesis test summarized (asymptotic significance) and significant (*p* < 0.05).

## 3. Results and Discussion

### 3.1. Total Phenolic Content and Antioxidant Capacity of Red Propolis

All the vacuum-packed and labeled sausage samples were stored at 2 °C in a digital BOD incubator until the respective analyses were conducted. The temperature was controlled and monitored to ensure the homogeneity and stability of the analysis.

The total phenolic value found in this study was 182.60 mg GAE/g for the red propolis extract and for sodium erythorbate, the value found was 695 mg GAE/g. Thus, the equivalence between the antioxidant capacity of the two antioxidants is 3.6 times (695/182.6), so the proposed treatments were 0.18% (1800 ppm—equivalent to erythorbate) and 0.36% (3600 ppm—twice as much as erythorbate), considering the maximum value of 0.05% (500 ppm) of sodium erythorbate added to the formulations.

When analyzing the ethanolic extract of red propolis, we found average values of 525.21 mg GAE/g, which were higher than those found in another study [29]. Refs [30] and [31] analyzed the antioxidant power using the Folin-Ciocalteu reagent and the methodology of Singleton et al. (1999) and found lower results than this study, 103.70 mg/g and 41.33 mg GAE/g, respectively. These variations may be due to the fact that the raw materials have different origins, compositions, extraction methods, and alternative solvents, as well as different geographical regions and times of the year [29,32].

Among the various methods used to determine antioxidant capacity, this study also used the DPPH and ABTS assays. The red propolis extract showed a high antioxidant capacity with DPPH and ABTS test values of 109,038 µg Trolox/g and 400.9 mmol Trolox/100 g of propolis, respectively. These values differ from the values found by [31], who found DPPH values of 180.00 μmol/Trolox/g propolis and ABTS values of 369.33 μmol Trolox/g propolis. It should be noted that there is a great deal of variation in the values found, mainly depending on the region of collection, time of year, flora, time of harvest, processing technique, and bee species [33,34].

### 3.2. Proximal Composition of Fresh Lamb Sausages

The results of the proximal composition are shown in Table 1. Similar values (*p* > 0.05) were observed for moisture and protein in all treatments. Differences were found (*p* < 0.05) in the parameters fat and ash, possibly due to variations in the meat raw materials, as no differences were expected between the treatments. The results obtained by [35] when adding mango extract powder to chicken sausage were similar in relation to fat and ash. However, [36], when adding tomato powder and linseed meal to beef sausages, obtained results with significant variations between treatments for ash, similar to those found in this study.

### 3.3. pH and Color Profile of Lamb Sausages

The effects of propolis extract and nitrite reduction on the pH of lamb sausages during 21 days of cold storage are shown in Table 2. In all treatments, there was a reduction (*p* < 0.05) in the pH value due to the microbial action that produces acids throughout the storage time. The sausages with antioxidants showed a gradual reduction in pH values, from pH 5.71 and pH 5.70 in P1N75 and P2N75, respectively, on day 7 to pH 5.24 in both treatments on day 21. There were no differences (*p* > 0.05) on day 21 between any of the treatments (5.29, 5.30, 5.24, and 5.24 in EN150, EN75, P1N75, and P2N75, respectively). The values obtained in this study differed from those reported by [37], who evaluated the influence of natural saffron extracts on the pH and shelf life of fresh lamb sausages and found pH values between 5.51 and 5.61.

The effects of propolis extract and nitrite reduction on the color of lamb sausages during 21 days of cold storage are shown in Table 3. With regard to the color profile, the storage time did not affect L* (*p* > 0.05). In particular, on day 7, treatments P1N75 and P2N75 showed the highest values (33.77 and 33.21, respectively), but with no statistical difference, suggesting that the antioxidant used in these treatments did not alter the brightness of the sausages. In the case of the EN150 and EN75 treatments, there was a significant difference in the L* values over the 21-day storage period, suggesting that the burgers had changed color.

For the color profile a*, there were significant differences (*p* < 0.05) in all treatments and during the storage days. On day 0, the a* values differed (*p* < 0.01) between EN150 (4.81) and P1N75 (2.91). This may have been due to the acceleration of the NO_2_ reaction with myoglobin caused by erythorbate in the EN150 and EN75 treatments, unlike the treatments (P1N75 and P2N75) without the addition of erythorbate, where lower a* values may have been due to the slower rate of nitrite reduction without erythorbate, even considering the red tone of the extract. However, there were increasing variations in all the treatments throughout evaluation days 7 and 14, and the values on day 21, at the end of storage, which reached EN150 (6.22), EN75 (6.62), P1N75 (7.07), and P2N75 (7.58), were higher when propolis was added, possibly due to the sum of the factors in the red propolis color and good oxidative stability of the pigment caused by the extract. In reports by [36,37,38], a* values decreased during storage when natural antioxidants were added, which does not agree with the values obtained in this study.

The addition of extract showed significant differences in the b* values (*p* < 0.05) in the EN75 and P1N75 treatments (Table 3) during storage, demonstrating the tendency towards a yellow/green color, which for meat products is not an attractive color for consumers. Thus, the EN150 and P2N75 treatments, where no b* values with significant differences were obtained, are treatments in which the yellowish color without variations during storage from 0 to 21 days meets the criterion of a color attribute attractive to consumers for consumption.

### 3.4. TBARS

The shelf life of meat products is limited by factors such as lipid oxidation [39]. Table 4 shows the increasing TBARS values of lamb sausages with the red propolis extract over time. The addition of antioxidants (*p* < 0.01) and storage time (*p* < 0.05) influenced the MDA content. On days 0, 7, and 14 of storage under refrigeration, no significant differences were observed between treatments. TBARS levels increased (*p* < 0.05) over time for all treatments, showing a similar behavior, decreasing on day 7 and increasing until the end of the storage time. On day 21, the EN150 batch showed the highest level (*p* < 0.05) of MDA, whereas the EN75 treatment and the sausages with the propolis extract showed better oxidative stability. This result shows the equalized antioxidant power between the synthetic antioxidant (sodium erythorbate) and the natural antioxidant (red propolis extract), even at the lowest concentration tested. It is worth noting that MDA values between 1 and 2 mg/kg are considered a sensory threshold for consumer perception [40]. Thus, despite the differences observed, all the treatments showed values that would not cause consumers to reject this attribute (0.20 to 0.81 mg MDA/kg) during the entire evaluation period in which the samples were refrigerated.

### 3.5. Index of Peroxide

**Figure 1:**
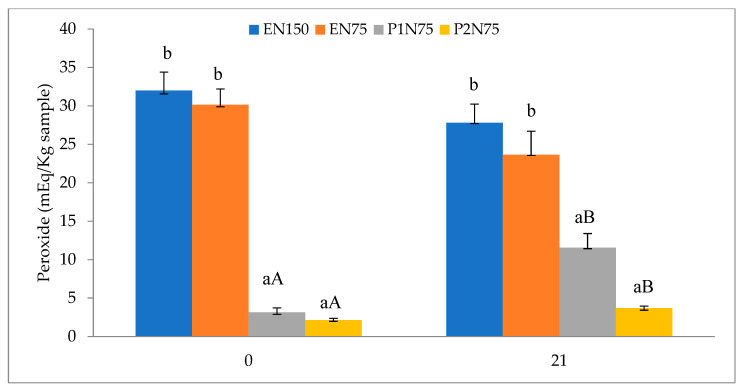
Peroxide index of fresh lamb sausages with reduced nitrite and added propolis extract, stored for 21 days at 2 °C. Different lowercase letters indicate a significant difference between treatments, and different uppercase letters indicate a significant difference between days. EN150 (control, BF + 500 mg/kg sodium erythorbate and 150 mg/kg sodium nitrite); EN75 (BF + 500 mg/kg sodium erythorbate and 75 mg/kg sodium nitrite); P1N75 (without the addition of erythorbate, BF + 1800 mg/kg propolis extract and 75 mg/kg sodium nitrite); and P2N75 (without the addition of erythorbate, BF + 3600 mg/kg propolis extract and 75 mg/kg sodium nitrite (Tukey’s test *p* < 0.05).

Figure 1 shows the peroxide index values of the fresh lamb sausages during storage for 21 days at 2 °C. The peroxidation results on day 0 showed differences (*p* < 0.001) between the treatments, with the peroxide indices found in the treatments with propolis extract being lower than those with sodium erythorbate (31.98 and 30.13 vs. 3.15 vs. 2.13 mEq/kg for com and EN75 vs. P1N75 and P2N75, respectively). In the EN150 and EN75 treatments, no significant differences were found between the times (0 and 21 days) evaluated, but the values found were high, exceeding the recommended level for human consumption (20 mEq/kg), according to [41]. In the P1N75 and P2N75 treatments, the peroxide index values increased (*p* < 0.01) from day 0 to day 21 but were much lower than EN150 and EN75. These results are promising, since propolis extract was added to these treatments, demonstrating the purpose of its use, which is to prevent lipid oxidation of fresh sausages during storage.

### 3.6. Texture

Texture profile analysis (TPA) is a crucial factor when it comes to assessing the quality of restructured meat products and is described in Table 5. The addition of the red propolis extract and the reduction in nitrite content did not cause any difference (*p* < 0.05) between the treatments in relation to all the textural parameters of the fresh sausage profile (Table 5). On the other hand, hardness decreased over time in the P1N75 treatment (*p* < 0.001) from 72.95 N to 55.78 N, and elasticity decreased in the EN150 treatment (*p* < 0.05) from 0.74 mm to 0.70 mm and P1N75 treatment (*p* < 0.01) from 0.74 mm to 0.65 mm. Chewiness was reduced in the EN150 treatment (*p* < 0.05) from 29.59 N.mm to 24.83 N.mm and in the P1N75 treatment (*p* < 0.001) from 33.00 N.mm to 22.28 N.mm. This corroborates the results of the work by [42], who used natural antioxidants in sausages and obtained values that decreased over the storage time. In other words, the incorporation of the red propolis extract did not cause negative changes in the texture profile of the fresh sausages, as the EN150 and P1N75 treatments showed similar values.

Weight loss to cooking (Table 5) was recorded in the range of 26.64–32.25% between treatments on day 0, which showed a significant difference (*p* < 0.01), with the highest value for treatment P1N75, lamb sausages with reduced nitrite and propolis extract. On day 21, cooking loss was recorded in the range of 26.89–34.01%, showing a significant difference between the treatments (*p* < 0.05) and with greater weight loss in the samples from the EN75 treatment. These samples have reduced nitrite and artificial antioxidants in the formulation, which is not a good indicator of the parameters that the industry is looking for to obtain a reduced WLC. This shows that the P1N75 treatment with reduced nitrite and added propolis extract is not significantly different from the EN150 treatment (maximum nitrite and no antioxidant), so it would better meet this criterion desired by industry and consumers. Reference [37] obtained WLC results between 21.16% and 26.94% (*p* < 0.05) in treatments with the addition of a natural turmeric antioxidant in lamb sausage, which were lower than those found in this study. Cooking loss causes the loss of fluids containing nutrients (water soluble) and pigments (color formers) [35] and can also be influenced by the ability of the protein matrix to stabilize and/or immobilize fat molecules [43]; thus, the meat of the animal species and the antioxidant used in meat products must be taken into account.

### 3.7. Residual Nitrite

**Figure 2:**
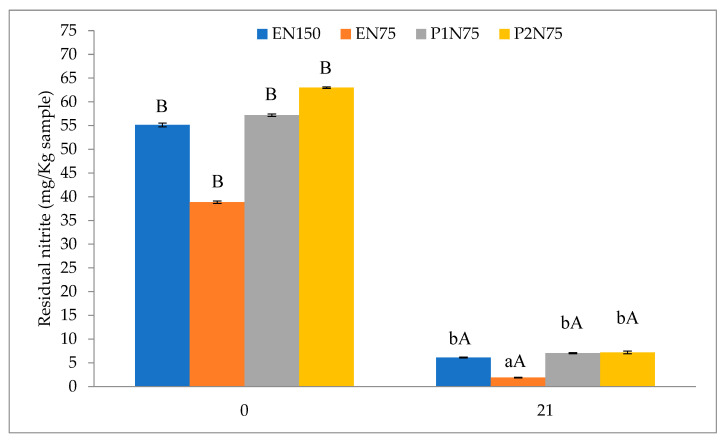
Residual nitrite in fresh lamb sausages with reduced nitrite and added propolis extract stored for 21 days at 2 °C. Error bars correspond to the standard error. Different lowercase letters indicate a significant difference between treatments, and different uppercase letters indicate a significant difference between days. EN150 (control, BF + 500 mg/kg sodium erythorbate and 150 mg/kg sodium nitrite); EN75 (BF + 500 mg/kg sodium erythorbate and 75 mg/kg sodium nitrite); P1N75 (without the addition of erythorbate, BF + 1800 mg/kg propolis extract and 75 mg/kg sodium nitrite); P2N75 (without the addition of erythorbate, BF + 3600 mg/kg propolis extract and 75 mg/kg sodium nitrite (Tukey’s test *p* < 0.05).

The residual nitrite found in the fresh sausages is shown in Figure 2. It can be seen that there was a significant difference (*p* < 0.01) on day 21 and in all treatments during the storage time. The values found on day 0 (55.41 vs. 38.84 vs. 57.20 vs. 62.00 mg/kg for EN150, EN75, P1N75, and P2N75, respectively) were higher, as expected, because on the first day, there was less time for the nitrite to react with myoglobin during storage. Thus, the values observed on day 21 (6.12 vs. 1.90 vs. 7.04 vs. 7.20 mg/kg for EN150, EN75, P1N75, and P2N75, respectively) are lower, because over time, the nitrite that was added to the formulations tended to decrease, turning into nitric oxide and therefore reacting with myoglobin to form myoglobin complexes, resulting in the characteristic color of cured meat products [44]. Results of nitrite reduction with storage time were observed by [36] when adding tomato and linseed powder to fresh beef sausages and [45], who evaluated residual nitrite in sausages with healthy fats replacing animal fat. In this experiment, when looking at the results by treatment, it is clear that the lowest value (1.90 mg/kg on day 21) was found in the EN75 treatment, which has chemical additives (sodium erythorbate) in its formulation to take part in the aforementioned reactions and reduce the initial nitrite content compared to EN150.

According to [46], the acceptable daily intake of nitrite is 0.4 mg/kg of body weight, which means that for an adult weighing 60 kg, it is recommended to ingest up to 24 mg of nitrite daily. It is important to note that Brazilian legislation [47] limits the use of sodium nitrite to 150 mg/kg in fresh sausages because it is known that when there are high levels of nitrite in the human body, it acts on hemoglobin by oxidizing iron, preventing hemoglobin from transporting oxygen [48]. This can be linked to increased blood pressure and even some types of cancer, such as gastrointestinal cancer, precisely because of the formation of nitrosamines, which are carcinogenic substances [49]. As seen in this study, with storage time, the residual nitrite value decreases; therefore, if the recommended legal limits are followed, it can be said that there is little or no risk to consumer health [50], even more so when natural products are present in the formulation, which help to reduce these risks to consumer health.

### 3.8. Free Fatty Acids

The content of free fatty acids (FFAs) present in lamb sausages is shown in Table 6. The release of fatty acids from molecules such as phospholipids and triglycerides is a phenomenon influenced by various factors, such as endogenous lipases, raw materials, processing conditions, additives, and ingredients [1]. In all treatments, the main free fatty acids found were monounsaturated fatty acids (MUFAs; 48.04–55.26%), followed by saturated fatty acids (SFAs; 34.13–40.63%) and polyunsaturated fatty acids (PUFAs; 9.15–11.68%) on days 0 and 21, respectively. When analyzing the fatty acids individually, it was observed that oleic acid was the predominant one (43.10%), followed by palmitic acid (20.70%), stearic acid (15.98%), and linoleic acid (9.03%). The other fatty acids were individually <12%. The amount of FFAs in all treatments showed the following relationship between treatments and FFAs: MUFAs < SFAs < PUFAs. This suggested a preferential release of MUFAs over other FFAs.

Total FFAs were similar between treatments (EN150, EN75, P1N75, and P2N75) on days 0 and 21, with no significant differences in most free fatty acids, with the exception of C14:0 (myristic acid), C16:0 (palmitic acid), and C18:2n-3 (linolenic acid). On day 0, C14:0 (myristic acid) showed a significant difference (*p* < 0.05) between the treatments and lower values in those (P1N75 and P2N75) where propolis extract was added; therefore, in the EN150 and EN75 treatments, the values found were higher. Over the 21 days, the values showed significant differences (*p* < 0.05) and a reduction in the values of this fatty acid in all treatments. On day 0, there was a significant difference between treatments in C16:0 (palmitic acid), and C18:2n-3 (linolenic acid) showed a significant difference (*p* < 0.05) only in EN75 from day 0 to day 21.

### 3.9. Microbiology Analysis

Propolis is known for its antibacterial action, which is attributed to its chemical composition rich in flavonoids (quercetin and naringenin) that promote changes in the bacterial membrane and its mobility [51]. Fresh sausages are highly perishable products, and this affects their stability and shelf life, as they have favorable conditions for the growth of microorganisms. The evaluations conducted for the presence of *Salmonella* spp. and *Escherichia coli* on days 0 and 21 were negative, proving the microbiological safety of the sausages evaluated. The results of the analysis of psychrotrophic aerobic microorganisms were affected by storage and the formulation (Table 7). The levels of psychrotrophs in all the treatments showed a gradual increase (*p* < 0.05). In other words, microbial growth was similar in all treatments, and there was no antimicrobial benefit from the addition of red propolis, as expected.

Monitoring psychrotrophic aerobic bacteria is especially important, as they can cause changes in smell, texture, and taste as a result of the production of different metabolic compounds when present in food [52]. Brazilian legislation lists this analysis as mandatory for the marketing of these products [53]. In the results of the experiments by [54] using an ethanolic extract of green propolis in beef burgers for 8 days of storage, they found higher psychrotrophic values (7.7 log_10_ CFU/g to 9.3 log_10_ CFU/g) than those found in this study, which were 6.39 log_10_ CFU/g to 6.76 log_10_ CFU/g. This difference may be due to the fact that the sausages in this experiment, despite being stored for 21 days, were vacuum-packed, contributing to less exposure to lipid oxidation, pH reduction, and, therefore, microbial growth.

### 3.10. Sensory Acceptance

The results of the sensory acceptance tests, including the attributes aroma, flavor, texture, juiciness, and overall acceptance, of the sausages containing propolis extract and nitrite after they had been roasted, prepared, and evaluated by 120 tasters are shown in Table 8. Juiciness, texture, flavor, and overall acceptability were the attributes that showed significant differences between the treatments. Lower scores (*p* < 0.05) for all attributes were recorded for sausages with a low percentage of the red propolis extract (P1N75), possibly due to the aftertaste that the propolis and lamb meat left in the sausage. In the case of P2N75, in which a higher percentage of propolis was incorporated, the texture (7.41) and juiciness (7.33) attributes did not differ significantly (*p* < 0.05) from the values attributed to EN75, which may suggest that consumers accept this product more readily than P1N75. These results indicate that this functional ingredient (propolis extract) can be incorporated into sausage products, resulting in acceptable products. The authors of [55], when analyzing the taste of lamb burgers containing the natural antioxidants oregano, rosemary, basil, sodium erythorbate, and lemon extract, obtained an overall average acceptance of 6.80 among all the treatments, which differed from the average score of 7.32 given in this work.

It is clear that the use of red propolis in lamb products is better accepted than that of other extracts, as the work by [55] shows. In relation to the sensory analysis conducted on cooked sausages, all groups received a score on the acceptance test higher than seven (I liked it moderately, according to the hedonic scale used in this study) for all attributes. In this context, it is clear that the use of propolis in sausages can be a natural antioxidant option with a healthier appeal and good sensory acceptance by consumers.

## 4. Conclusions

The results obtained in this study suggest that the addition of red propolis extract improved the antioxidant status of lamb sausages. Reducing the sodium nitrite content did not increase lipid oxidation or harm the microbiological characteristics of the sausages. The medium level of extract addition (0.18%) caused fewer changes in the sausages’ color and texture parameters than the other treatments. Therefore, these findings open up prospects for the application of propolis extract as a natural antioxidant in meat products without altering the physicochemical properties during the storage period.

## Figures and Tables

**Table 1:** Proximate composition of lamb sausages with reduced nitrite and added propolis extract, stored for 21 days at 2 °C.

Composition (%)	EN150	EN75	P1N75	P2N75	SE	Sig.
Moisture	63.17	67.07	64.49	64.81	0.2368	ns
Fat	15.19 ^b^	14.67 ^b^	12.52 ^a^	13.12 ^a^	0.2573	*
Protein	17.82	18.08	18.57	18.27	0.1416	ns
Ash	2.22b	2.19 ^ab^	2.13 ^ab^	2.04 ^a^	0.0219	*

^a,b^: Mean values on the same line with different letters indicate a significant difference. SE: Standard error; Sig.: significant; ns: not significant; * *p* < 0.05 (Tukey’s test). EN150 (control, BF + 500 mg/kg sodium erythorbate and 150 mg/kg sodium nitrite); - EN75 (BF + 500 mg/kg sodium erythorbate and 75 mg/kg sodium nitrite); - P1N75 (without the addition of erythorbate, BF + 1800 mg/kg propolis extract and 75 mg/kg sodium nitrite); - P2N75 (without the addition of erythorbate, BF + 3600 mg/kg propolis extract and 75 mg/kg sodium nitrite).

**Table 2:** Effect of propolis extract on the pH parameters of fresh lamb sausages during storage at 2 °C.

Treatments							
	Days	EN150	EN75	P1N75	P2N75	SE	Sig.
pH	0	5.84 ^A^	5.83 ^A^	5.81 ^A^	5.81 ^A^	0.0072	ns
	7	5.85 ^bA^	5.80 ^bA^	5.71 ^aB^	5.70 ^aB^	0.0106	*
	14	5.46 ^B^	5.37 ^B^	5.42 ^C^	5.39 ^C^	0.0271	ns
	21	5.29 ^B^	5.30 ^B^	5.24 ^D^	5.24 ^D^	0.0112	ns
	SE	0.0135	0.0117	0.0090	0.0085	-	-
	Sig.	***	**	**	***	-	-

^a,b^: Average values in the same row with different letters indicate a significant difference. ^A–D^: Average values in the same column with different letters indicate a significant difference. SE: Standard error; Sig.: significant; ns: not significant; * *p* < 0.05, ** *p* < 0.01, *** *p* < 0.001 (Tukey’s test).

**Table 3:** Effect of propolis extract on the color profile of fresh lamb sausages during storage at 2 °C.

Treatments							
	Days	EN150	EN75	P1N75	P2N75	SE	Sig.
L*	0	34.00 ^AB^	34.75 ^B^	33.79	34.65	0.4695	ns
	7	31.16a ^bA^	29.50 ^aA^	33.77 ^b^	33.21 ^b^	0.4579	*
	14	34.57 ^B^	33.55 ^B^	33.28	36.07	0.4653	ns
	21	35.65 ^B^	34.79 ^B^	34.81	35.68	0.3505	ns
	SE	0.4521	0.4398	0.4550	0.4057	-	-
	Sig.	*	**	ns	ns	-	-
a*	0	4.81 ^bA^	4.27 ^bA^	2.91 ^aA^	3.82 ^abA^	0.1486	**
	7	5.55 ^abAB^	5.33 ^abA^	4.75 ^aB^	6.15 ^bB^	0.1611	*
	14	6.21 ^abB^	6.75 ^bB^	5.46 ^aB^	6.74b ^BC^	0.1652	*
	21	6.22 ^aB^	6.62 ^abB^	7.07 ^abC^	7.58 ^bC^	0.1562	*
	SE	0.1660	0.1556	0.1600	0.1495	-	-
	Sig.	*	*	**	**	-	-
b*	0	8.33	8.16 ^B^	7.88 ^AB^	8.93	0.2146	ns
	7	7.73 ^ab^	6.74 ^aAB^	8.28 ^bB^	8.90 ^b^	0.1784	*
	14	8.12	7.49 ^B^	7.42 ^AB^	8.26	0.1763	ns
	21	6.93 ^ab^	5.88 ^aA^	6.74 ^abA^	7.74 ^b^	0.1783	**
	SE	0.1955	0.1864	0.197	0.1701	-	-
	Sig.	**	***	**	**	-	-

^a,b^: Average values in the same row with different letters indicate a significant difference. ^A–C:^ Average values in the same column with different letters indicate a significant difference. SE: Standard error; Sig.: significant; ns: not significant; * *p* < 0.05, ** *p* < 0.01, *** *p* < 0.001 (Tukey’s test).

**Table 4:** Effect of propolis extract on TBAR parameters of fresh lamb sausages during storage at 2 °C.

Treatments							
	Days	EN150	EN75	P1N75	P2N75	SE	Sig.
TBARs	0	0.29 ^A^	0.33 ^A^	0.28 ^A^	0.36 ^B^	0.0123	ns
	7	0.22 ^A^	0.26 ^A^	0.20 ^A^	0.24 ^A^	0.0123	ns
	14	0.36 ^A^	0.43 ^B^	0.37 ^AB^	0.39 ^B^	0.0115	ns
	21	0.81 ^bB^	0.51 ^aB^	0.41 ^aB^	0.39 ^aB^	0.0355	*
	SE	0.0343	0.0123	0.0148	0.0121	-	-
	Sig.	**	***	**	**	-	-

^a,b^: Mean values in the same row with different letters indicate a significant difference. ^A,B:^ Mean values in the same column with different letters indicate a significant difference. SE: Standard error; Sig.: significant; ns: not significant; * *p* < 0.05, ** *p* < 0.01, *** *p* < 0.001 (Tukey’s test).

**Table 5:** Effect of red propolis on the texture profile and weight loss on cooking (WLC) of lamb sausages during storage at 2 °C for 21 days.

Treatments							
	Days	EN150	EN75	P1N75	P2N75	SE	Sig.
Hardness (N)	0	66.07	66.43	72.95 ^B^	65.23	1.2211	ns
	21	61.48	64.00	55.78 ^A^	61.11	1.2273	ns
	SE	1.8429	2.0135	1.6753	1.3163	-	-
	Sig.	ns	ns	***	ns	-	-
Elasticity	0	0.74	0.74 ^B^	0.74 ^B^	0.73	0.0110	ns
(mm)	21	0.70	0.67 ^A^	0.65 ^A^	0.70	0.0080	ns
	SE	0.0111	0.0176	0.0109	0.0140	-	-
	Sig.	ns	*	**	ns	-	-
Cohesiveness	0	0.52	0.52	0.53	0.52	0.0056	ns
	21	0.50	0.53	0.54	0.53	0.0060	ns
	SE	0.0079	0.0098	0.0085	0.0064	-	-
	Sig.	ns	ns	ns	ns	-	-
Chewability	0	29.59 ^B^	30.21	33.00 ^B^	29.40	0.8690	ns
(N. mm)	21	24.83 ^A^	25.78	22.28 ^A^	26.40	0.6278	ns
	SE	0.9195	1.3132	0.8979	1.1050	-	-
	Sig.	*	ns	***	ns	-	-
Weight loss	0	26.64 ^a^	28.87 ^abA^	32.25 ^b^	26.96 ^aA^	0.4991	**
To cooking	21	26.89 ^a^	34.01 ^bB^	31.57 ^a^	33.65 ^bB^	0.7663	*
(%)	SE	0.4838	1.1205	0.9867	0.9394	-	-
	Sig.	ns	*	ns	**	-	-

^a,b^: Mean values in the same row with different letters indicate a significant difference. ^A,B^: Mean values in the same column with different letters indicate a significant difference. EP: Standard error; Sig.: significant; ns: not significant; (Tukey’s test) * *p* < 0.05, ** *p* < 0.01, *** *p* < 0.001.

**Table 6:** Free fatty acids in lamb sausages prepared with erythorbate nitrite (EN150), reduced nitrite and erythorbate (EN75), and reduced nitrite and red propolis extract (P1N75 and P2N75) stored at 2 °C.

Free Fatty Acids		Treatments					
Mean Peak Area (%)	Day	EN150	EN75	P1N75	P2N75	SE	Sig.
C14:0	0	2.26 ^b^	1.87 ^abA^	1.65 ^a^	1.94 ^ab^	0.09	*
(myristic acid)	21	2.12 ^ab^	2.18 ^bB^	1.81 ^ab^	1.75 ^a^	0.09	*
	Sig.	ns	*	ns	ns	-	-
C16:0	0	23.30 ^b^	18.52 ^a^	19.03 ^ab^	20.95 ^ab^	1.10	*
(palmitic acid)	21	21.32	21.48	20.07	20.99	1.10	ns
	Sig.	ns	ns	ns	ns	-	-
C16:1n-7	0	3.93	3.79	3.46	4.02	0.34	ns
(palmitoleic acid)	21	3.86	3.62	3.71	4.26	0.34	ns
	Sig.	ns	ns	ns	ns	-	-
C18:0	0	15.06	18.84	13.44	15.49	1.54	ns
(Stearic acid)	21	16.93	15.21	16.43	16.00	1.54	ns
	Sig.	ns	ns	ns	ns	-	-
C18:1n-9c	0	41.45	42.57	47.81	42.36	1.90	ns
(oleic acid)	21	43.22	41.61	43.97	41.85	1.90	ns
	Sig.	ns	ns	ns	ns	-	-
C18:1n-9t	0	2.66	3.19	4.00	4.08	0.64	ns
(elaidic acid)	21	3.00	2.86	3.33	3.81	0.64	ns
	Sig.	ns	ns	ns	ns	-	-
C18:3n-6	0	9.11	9.73	8.80	9.32	0.57	ns
(α-linolenic acid)	21	8.20	8.06	9.28	9.78	0.57	ns
	Sig.	ns	ns	ns	ns	-	-
C18:3n-3	0	2.23 ^B^	1.51	1.82	1.83	0.31	ns
(linolenic acid)	21	1.17 ^A^	1.09	1.40	1.55	0.31	ns
	Sig.	*	ns	ns	ns	-	-
SFAs	0	40.63	39.23	34.13	38.38	1.64	ns
	21	40.36	38.85	38.31	38.74	1.64	ns
	Sig.	ns	ns	ns	ns		-
MUFAs	0	48.04	49.56	55.26	50.47	2.19	ns
	21	50.08	48.08	51.01	49.92	2.19	ns
	Sig.	ns	ns	ns	ns	-	-
PUFAs	0	11.33	11.24	10.61	11.15	0.70	ns
	21	9.38	9.15	10.68	11.34	0.70	ns
	Sig.	ns	ns	ns	ns	-	-

^a,b^: Mean values in the same row with different letters indicate a significant difference. ^A,B^: Mean values in the same column with different letters indicate a significant difference. SE: Standard error; Sig.: significant; ns: not significant; (Tukey’s test) * *p* < 0.05.

**Table 7:** Changes in the total count of psychrotrophic microorganisms (CTP – log_10_ CFU/g) in lamb sausages with natural antioxidants during the storage period at 2 °C.

Total Psychrotrophic Count (CTP- log_10_ UFC/g)	EN150	EN75	P1N75	P2N75	SE	Sig.
Day 0	4.39 ^A^	4.24 ^A^	4.32 ^A^	4.22 ^A^	0.51	ns
Day 7	4.67 ^A^	4.34 ^A^	5.07 ^AB^	4.98 ^AB^	0.52	ns
Day 14	5.60 ^AB^	5.92 ^AB^	6.10 ^AB^	6.17 ^AB^	0.51	ns
Day 21	6.83 ^B^	6.68 ^B^	6.40 ^B^	6.76 ^B^	0.52	ns
SE	0.51	0.52	0.51	0.52	-	-
Sig.	*	*	*	*	-	-

^A,B^: Average values in the same column with different letters indicate a significant difference. Sig.: significant; ns: non-significant; SE: Standard error (Tukey’s test, * *p* < 0.05).

**Table 8:** Acceptance scores according to the preference of consumers on day 0 of cooked lamb sausage.

Acceptance Test	EN150	EN75	P1N75	P2N75	SE	Sig.
Aroma	7.37	7.06	7.06	7.06	0.114	ns
Texture	7.63 ^b^	7.37 ^ab^	7.21 ^a^	7.41 ^ab^	0.102	*
Flavor	7.74 ^b^	7.43 ^ab^	7.08 ^a^	7.17 ^a^	0.105	*
Juiciness	7.61 ^b^	7.42 ^ab^	7.21 ^a^	7.33 ^ab^	0.380	*
Overall acceptance	7.66 ^b^	7.35 ^ab^	7.16 ^a^	7.13 ^a^	0.108	*

^a,b^: Mean values on the same line with different letters indicate a significant difference. Sig.: significant; ns: not significant; SE: standard error (Tukey’s test, * *p* < 0.05). Sausages prepared (EN150) with sodium erythorbate and nitrite (EN75) or red propolis extract and nitrite (P1N75 and P2N75). Hedonic scale used: 1 = disliked very much; 2 = disliked very much; 3 = disliked; 4 = disliked slightly; 5 = neither liked nor disliked; 6 = liked slightly; 7 = liked regularly; 8 = liked very much; and 9 = liked very much.

## Data Availability

Data are contained within the article.

## Abbreviations

ABTS	2,2′-Azino-bis (3-ethylbenzothiazolin) 6-acidosulfonic acid
AOAC	Association of Official Agricultural Chemists
BOD	Biochemical oxygen demand
CAAE	Certificate of Presentation of Ethical Review
CFU	Colony forming unit
CTP	Total psychrotrophic count
DPPH	1,1-diphenyl-2-picrylhydrazyl
FFAs	Free fatty acids
FZEA	School of Animal Science and Food Engineering
GAE	Gallic acid equivalent
MDA	Malonaldehyde
MUFAs	Monounsaturated fatty acids
PUFAs	Polyunsaturated fatty acids
SAS	Statistical Analysis System
SFAs	Saturated fatty acid
TBARS	Thiobarbituric acid
TPA	Texture profile
TEP	1,1,3,3-Tetraethoxypropane
USP	University of São Paulo
WLC	Weight loss to cooking
